# Identifying Drivers of Nonconsensual Intimate Image Sharing: A Mixed-Methods Synthesis of Perpetrator Research

**DOI:** 10.1177/15248380251375912

**Published:** 2025-10-04

**Authors:** Stine Nygård, Maren Kristin Nysæter, Ligia Ribeiro Ferreira, Lars Roar Frøyland, Ingela Lundin Kvalem

**Affiliations:** 1 University of Oslo, Norway; 2 Federal University of Pernambuco (UFPE), Brazil; 3 OsloMet—Oslo Metropolitan University, Norway

**Keywords:** non-consensual sharing of intimate images, perpetrator characteristics, perpetrator motives, social dynamics, mixed-methods research synthesis

## Abstract

Echoing the many and serious consequences of nonconsensual sharing of intimate images (NCSII), calls for preventive measures have reverberated across public, legal, and academic discourses. However, at present, empirical insights that could aid the development of perpetrator-focused interventions remain fragmented. Responding to this predicament, we carried out a mixed-methods research synthesis through which we aimed to identify and distill all extant research on the psychosocial mechanisms of NCSII, focusing in particular on perpetrator motives and characteristics, as well as the social dynamics of NCSII incidents. A systematic search across six databases (Web of Science, Scopus, PsycInfo, PubPsych, PubMed, and ERIC) yielded 10,324 unique records, of which 68 peer-reviewed articles were deemed eligible following screening and full-text evaluation. Findings from quantitative (*n* = 55) and qualitative (*n* = 13) studies were synthesized separately and later integrated in an overarching discussion. Through this juxtaposition, we found that NCSII appears to primarily transpire as a form of homosocial bonding, initiated by individuals who tend to have more experiences with intimate sharing practices than non-perpetrators, and whose actions are situated in a wider social context of hegemonic masculinity, female sexual objectification, and sexism. Notably, although gender was not found to be a reliable predictor of perpetration, our synthesis indicates that the social dynamics of NCSII differ for boys/men and girls/women, underscoring the need for gender-sensitive approaches in both research and prevention.

Over the past decade, nonconsensual sharing of intimate images (NCSII) has grown increasingly prevalent and been consistently linked to a myriad of negative outcomes (Schmidt et al., 2024). In recognition of this, calls for preventive measures have reverberated across public, legal, and academic discourses, with initial efforts focusing on discouraging young people from sharing intimate images of themselves in the first place (e.g., EPICTUK, 2011). This approach, however, has been widely criticized for being ineffective and placing responsibility and blame on victims (e.g., Dobson & Ringrose, 2016). As an alternative, researchers in the field have suggested that it may be more constructive to target the actions of perpetrators (e.g., Gavey et al., 2024), which requires an in-depth understanding of the social and psychological drivers behind the behavior in question (Michie et al., 2011).

However, current insights into NCSII perpetration remain limited, largely due to the fragmented nature of the research landscape. For instance, numerous terms have been used to denote the violation, including sexting (e.g., Krieger, 2017), sexual cyberbullying (e.g., Ehman & Gross, 2019), revenge/nonconsensual pornography (e.g., Walker & Sleath, 2017), image-based sexual abuse (Henry & Beard, 2024), and technology-facilitated sexual violence (Henry & Powell, 2018). Notably, these varying conceptualizations tend to either limit the focus to specific forms of NCSII occurring under specific circumstances (e.g., revenge after break-up; for a discussion, see McGlynn et al., 2017) or conflate NCSII with theoretically related, though distinct, actions (e.g., voluntarily sending intimate images of oneself to another person; for a discussion, see Krieger, 2017). While such approaches may be useful for contextualizing NCSII as a part of broader practices and forms of violence, they risk obscuring empirical insights into the potentially unique mechanisms underlying NCSII perpetration, which may be relevant to the development of preventive measures.

Against this backdrop, the present review aims to distill extant knowledge about the psychosocial mechanisms of NCSII perpetration, specifically. To circumvent the above issues, we draw on all available peer-reviewed research on nonconsensual sharing of intimate images, regardless of the terminology utilized by the relevant studies. In this context, we use “nonconsensual” to refer to the nature of perpetrators’ actions, which are generally assumed to be carried out against the will or without the knowledge of their victims. By “sharing,” we mean any way of giving other people access to the image, including showing it in person, sending it via messaging services, and posting images on websites, social media, or online forums. With “intimate,” we include images that contain nudity but also acknowledge that images may be perceived as sexual without depicting sex acts or reproductive organs. Finally, the term “images” includes both photos and videos, whether real, manipulated, or fabricated.

Although a number of literature reviews and meta-analyses have already touched on the topic of NCSII, these have tended to focus on broader issues, such as sexting (e.g., Barroso et al., 2023), image-based sexual abuse (e.g., Paradiso et al., 2024), and technology-facilitated sexual violence (e.g., Henry & Powell, 2018). Due to the broad scope of these syntheses, research on NCSII in general, and perpetrators specifically, has only been briefly mentioned. One notable exception is Henry and Beard’s (2024) recently published scoping review on image-based sexual abuse perpetration—defined as the nonconsensual creation and threatened or actual dissemination of intimate imagery—which included 21 studies that examined the motives and/or characteristics of NCSII perpetrators.

While the aim of Henry and Beard’s (2024) review parallels that of the present synthesis, there are some important distinctions between our respective contributions to the field. While their review was the first to focus exclusively on image-based sexual abuse perpetration, ours is the first to solely synthesize research on NCSII perpetration. As such, the thematic scope of this review is narrower, allowing a more detailed synthesis of research findings. At the same time, our search string included a greater variety of terms used to refer to NCSII in the literature, resulting in the inclusion of more than three times as many studies on NCSII perpetration, compared to Henry and Beard’s (2024) review. Hence, this article seeks to present a more comprehensive synthesis of the current body of knowledge.

We conducted the review in line with Heyvaert et al.’s (2017) framework for *mixed-methods research synthesis* (MMRS), which is a type of integrative review that synthesizes quantitative and qualitative research on complementary or overlapping research questions using systematic methods. As with primary mixed-methods studies, the main strength of MMRS is that it enables critical analysis of convergences and divergences of quantitative and qualitative research findings. Essentially, it consists of two strands of research (one qualitative and one quantitative) that are integrated through a joint presentation or discussion of findings. In keeping with this approach, we devised the following research questions: (a) What have quantitative studies found regarding the motives and characteristics of NCSII perpetrators? (b) What have qualitative studies found regarding the motives for, and social dynamics of, NCSII perpetration? (c) What do the convergences and divergences between these bodies of knowledge indicate regarding the driving forces of NCSII and current research gaps?

## Method

### Study Design

This review followed the Preferred Reporting Items for Systematic Reviews and Meta-Analyses (PRISMA; Moher et al., 2009). In line with Heyvaert et al.’s (2017) guidelines for segregated MMRS, we initially selected, evaluated, and synthesized quantitative and qualitative studies separately, and later integrated key insights into an overarching discussion. We favored this approach over a fully integrative framework because it enabled us to review qualitative and quantitative findings in a way that resonated with the original research methods, and elucidate the distinct contributions by, and gaps within, each body of knowledge.

### Inclusion and Exclusion Criteria

To be included in the review, studies had to (a) report primary research on NCSII perpetration, (b) be published in a peer-reviewed journal, and (c) be written in English. In addition to this, quantitative studies were required to (d) report analyses of psychosocial covariates or self-reported motives of NCSII perpetration, while qualitative studies needed to (e) state or demonstrate that they had analyzed first-person data from NCSII perpetrators concerning the motives for, or social dynamics of, their transgressions. Studies were excluded if their primary focus was the production and/or dissemination of child sexual abuse material, or if their analyses conflated NCSII perpetration with other behaviors (e.g., various forms of sexting or image-based sexual abuse). However, studies that covered broader topics but reported separate analyses of NCSII perpetration were included in the review. As no previous reviews have focused exclusively on NCSII perpetration, we did not specify any cutoff dates regarding the publication years of the included studies.

### Literature Search

As the literature lacks an established and widely accepted term for NCSII, we strived to ensure that our search strategy was inclusive with regard to terminology. Hence, we constructed a three-part search string in which the first sequence included terms that have been coined for NCSII and related offenses, the second combined terms referring to the online sphere with synonyms for sexual aggression, and the third combined terms referring to intimate imagery with terms for the abuse of such material:

(“image-based sexual abuse” OR “nonconsensual porn*” OR “non-consensual porn*” OR “revenge porn*” OR “deepfake porn*” OR “sexual assault image*” OR cyberviolence OR sextortion OR “sexual cyberbullying” OR upskirting OR creepshot*) OR ((digital OR online OR cyber OR technology OR internet) AND (“sexual harassment” OR “sexual abuse” OR “sexual violence” OR “dating abuse” OR “dating violence” OR “intimate partner violence”)) OR ((sexting OR “sex* image*” OR “nude image*” OR “intimate image*”) AND (forward* OR shar* OR disseminat* OR “non-consensual” OR nonconsensual))

The initial literature search was carried out on November 29, 2021, using Web of Science, Scopus, PsycInfo, PubPsych, and PubMed. These databases were selected for the purpose of covering a wide range of disciplines within and bordering the social sciences. Due to project delays, two subsequent searches were carried out on November 30, 2022, and May 29, 2024. For these additional searches, the database ERIC was included to identify relevant articles from educational journals not indexed in the previously utilized databases. To reduce the number of duplicate records, each of the additional literature searches (with the exception of the initial search in ERIC) was limited to articles published during or after the year of the previous literature search. The literature searches resulted in a total of 12,958, 5,823, and 6,742 records, respectively. All records were imported into EndNote and deduplicated using Bramer et al.’s (2016) method. A PRISMA flowchart of the review process is presented in Figure 1.

**Figure 1.**
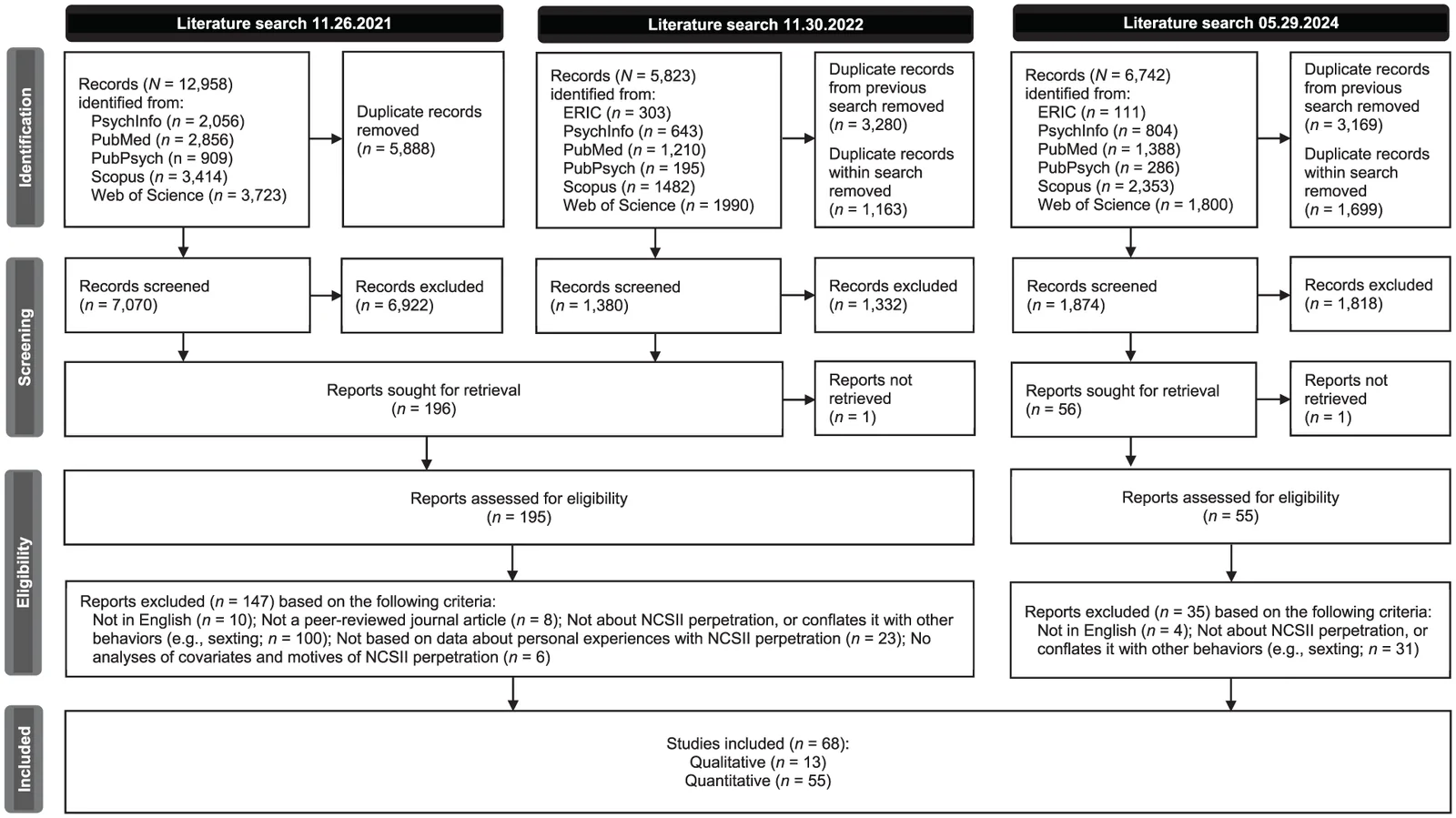
PRISMA flowchart of the literature search, screening, and selection process.

### Screening and Selection

In total, we identified 10,324 unique records. Titles and abstracts identified in the first and second literature searches (*n* = 8,450) were double-screened by two members of the review team using Rayyan, a web-based tool designed to aid the systematic review process. This led to the exclusion of 8,254 articles, with an inter-rater agreement of 95.6%. Next, potentially eligible records (*n* = 196) were retrieved (*n* = 195) and subjected to full-text review by the same pair of reviewers (inter-rater agreement = 74.8%), resulting in the inclusion of 48 articles. Records identified through the third literature search (*n* = 1,874) were screened by the main author alone, who excluded 1,818 articles based on titles and abstracts. Following full-text review of the remaining, retrievable records (*n* = 55), 20 articles from the third and final literature search were included. Of the 68 articles included in this review, 55 reported quantitative and 13 reported qualitative studies.

### Quality Assessment

We used the Mixed Methods Appraisal Tool (Hong et al., 2018, 2019) to identify common quality issues across studies. In line with Hong et al.’s (2018) instructions, we recorded our assessments of the quality criteria as “yes,” “no,” or “can’t tell,” and did not calculate an overall quality score or exclude studies based on this evaluation.

### Data Extraction, Coding, and Synthesis

We extracted descriptive data concerning the research aim(s) and question(s), setting(s), sample characteristics, and design of all included studies. For quantitative studies, we also extracted information about variables, measures, statistical procedures, and results from (a) bivariate and multiple regression analyses of psychosocial covariates of NCSII perpetration, and (b) univariate and gender comparative bivariate analyses of motives reported by NCSII perpetrators. For qualitative studies, we extracted information about theoretical frameworks, analytical approaches, and results concerning statements by, and interactions between, NCSII perpetrators. Due to difficulties in distinguishing first-person data from researchers’ interpretations, both were extracted for this review.

Both quantitative and qualitative findings were analyzed using NVivo. To address our first research question, results concerning perpetrator characteristics were coded using a vote-counting approach (Heyvaert et al., 2017), while results concerning motives were coded semantically (for details, see the Supplemental Materials). In synthesizing the findings for each construct, we generally adhered to the terminology used in the respective primary studies. However, in some instances, we have drawn upon widely recognized umbrella terms to denote psychological variables that were labeled differently across studies, but appeared to measure the same underlying constructs (e.g., cognitions). Importantly, constructs analyzed by one study only were ultimately excluded as results could not be meaningfully synthesized. To address our second research question, findings concerning the psychosocial dynamics of NCSII perpetration were analyzed using thematic synthesis (Thomas & Harden, 2008), which involved (a) coding data extracts inductively and generating (b) descriptive and (c) analytical themes (for an example of this process, see the Supplemental Materials).

## Results

### Quantitative Studies

We identified 55 studies examining the characteristics of perpetrators, of which six also explored their motives for engaging in NCSII. Summary tables of the key characteristics, findings, and quality assessments of all studies can be found in the Supplemental Materials.

In short, the included studies were published between 2013 and 2024, and presented findings based on cross-sectional (*n* = 52) and longitudinal (*n* = 3) data, collected from a total of 127,728 participants (ages 11–97 years) from the United States (*n* = 12), Italy (*n* = 7), Spain (*n* = 7), Belgium (*n* = 5), Canada (*n* = 4), Australia (*n* = 3), Portugal (*n* = 2), Brazil (*n* = 1), England (*n* = 1), Greece (*n* = 1), Netherlands (*n* = 1), Switzerland (*n* = 1), and various cross-national samples (*n* = 10). Prevalence rates of NCSII perpetration ranged from 0.4% (Gamez-Guadix et al., 2022) to 31.3% (Sesar et al., 2021). In part, this variation may be attributable to the wide range of instruments used to measure this transgression, which varied with regard to (a) how the perpetrator gained access to the image, (b) who the image portrayed, (c) what the image contained, (d) how the image was shared, and (e) whether it was specified that this act was nonconsensual. In terms of quality, the most frequently noted issues were studies’ use of non-representative samples and a lack of transparency about non-response bias.

#### Perpetrator Characteristics

Table 1 presents vote counts for the reported covariates of NCSII perpetration.

**Table 1.** Summary of Vote Counts for Reported Covariates of NCSII Perpetration.

Covariates	Count	References
Sociodemographic characteristics		
Gender		
Male > female	28	Agueli et al. (2024); Badenes-Ribera et al. (2024); Barrense-Dias et al. (2020); Barroso et al. (2021); Bianchi et al. (2016); Blais et al. (2025); Boer et al. (2021); Casas et al. (2019); Garcia et al. (2016); Kokkinos et al. (2024); Martínez-Bacaicoa et al. (2024); Morelli et al. (2016, 2020, 2021, 2023, 2024); Ojeda et al. (2019, 2024); Patchin and Hinduja (2019); Peskin et al. (2013); Powell et al. (2019, 2022); Ruvalcaba and Eaton (2020); Sesar et al. (2021); Silva et al. (2020); Strassberg et al. (2013, 2014)^a^; Wachs et al. (2021)
Nonsignificant	21	Clancy et al. (2019, 2020, 2023); Clancy, Klettke, et al. (2021); Clancy, Maas, et al. (2021); Gámez-Guadix et al. (2022, 2023); Holt et al. (2021); Hu et al. (2023); Karasavva & Forth (2022); Klein and Cooper (2019); Linares et al (2021); Maes et al. (2023); Said and McNealy (2023); Schokkenbroek et al. (2023); Seto et al. (2023); Strassberg et al. (2013, 2017)^a^; Van den Eynde et al. (2023); Van Ouytsel et al. (2019, 2021); Walker et al. (2021)
Age		
Nonsignificant	19	Barrense-Dias et al. (2020); Bianchi et al. (2016); Blais et al. (2025); Clancy et al. (2019, 2020, 2023); Clancy, Klettke, et al. (2021); Clancy, Maas, et al. (2021): Garcia et al. (2016); Hu et al. (2023); Klein and Cooper (2019): Martínez-Bacaicoa et al. (2024); Morelli et al. (2020)^a^; Morelli et al. (2023, 2024); Ojeda et al. (2024); Said and McNealy (2023); Schokkenbroek et al. (2023); Seto et al. (2023); Silva et al. (2020)
Positive association	10	Badenes-Ribera et al. (2024); Barroso et al. (2021); Gámez-Guadix et al. (2022, 2023); Maes et al. (2023); Ojeda et al. (2019); Patchin and Hinduja (2019)^a^; Van den Eynde et al. (2023)^a^; Van Ouytsel et al. (2019, 2021)
Negative association	7	Boer et al. (2021); Morelli et al. (2016, 2020, 2021)^a^; Patchin and Hinduja (2019)^a^; Ruvalcaba and Eaton (2020); Van den Eynde et al. (2023)^a^
Sexual orientation		
Nonsignificant	14	Bianchi et al. (2016); Blais et al. (2025); Boer et al. (2021); Garcia et al. (2016); Karasavva and Forth (2022); Klein and Cooper (2019); Kokkinos et al. (2024); Maes et al. (2023); Morelli et al. (2016); Seto et al. (2023); Silva et al. (2020); Van Ouytsel et al. (2019, 2021); Wachs et al. (2021)
Non-heterosexual > heterosexual	7	Badenes-Ribera et al. (2024); Barrense-Dias et al. (2020); Martínez-Bacaicoa et al. (2024); Morelli et al. (2020, 2023); Patchin and Hinduja (2019); Powell et al. (2019)
Ethnicity/race		
Nonsignificant	8	Blais et al. (2025); Klein & Cooper (2019)^a^; Patchin and Hinduja (2019); Peskin et al. (2013); Said and McNealy (2023); Seto et al. (2023); Silva et al. (2020); Wachs et al. (2021)
Non-white > white	1	Klein and Cooper (2019) ^a^
Non-western > western	1	Boer et al. (2021)
Foreign-born > not foreign-born	1	Barrense-Dias et al. (2020)
Relationship status		
Nonsignificant	5	Blais et al. (2025); Klein and Cooper (2019); Said and McNealy (2023); Sesar et al. (2021); Silva et al. (2020)
Nationality		
Significant^b^	4	Clancy, Klettke, et al. (2021)^a^; Morelli et al. (2020)^a^; Morelli et al. (2023); Powell et al. (2022)
Nonsignificant	2	Clancy, Klettke, et al. (2021)^a^; Morelli et al. (2020)^a^
Level of education		
Nonsignificant	1	Klein and Cooper (2019)
Negative association	2	Barrense-Dias et al. (2020); Boer et al. (2021)
Positive association	1	Maes et al. (2023)
Area of residence		
Rural > urban	1	Boer et al. (2021)
Urban > rural	1	Seto et al. (2023)
Nonsignificant	1	Barrense-Dias et al. (2020)
Socioeconomic status		
Nonsignificant	2	Barrense-Dias et al. (2020); Barroso et al. (2021)
Intimate image-sharing experiences		
NCSII victimization		
Positive association	14	Badenes-Ribera et al. (2024); Barroso et al. (2021); Boer et al. (2021); Clancy et al. (2020, 2023); Clancy, Klettke, et al. (2021); Clancy, Maas, et al. (2021); Gámez-Guadix et al. (2022); Karasavva and Forth (2022); Martínez-Bacaicoa et al. (2024); Said and McNealy (2023); Seto et al. (2023); Sparks et al. (2023); Van den Eynde et al. (2023); Walker et al. (2021)
Receiving NCSII		
Positive association	9	Badenes-Ribera et al. (2024); Barrense-Dias et al. (2020); Casas et al. (2019); Clancy et al. (2019, 2020, 2023); Clancy, Klettke, et al. (2021); Clancy, Maas, et al. (2021); Ojeda et al. (2019); Schokkenbroek et al. (2023)
Previous NCSII perpetration		
Positive association	3	Casas et al. (2019); Gámez-Guadix et al. (2022); Ojeda et al. (2019)
Sending sexts		
Positive association	16	Badenes-Ribera et al. (2024); Barrense-Dias et al. (2020); Casas et al. (2019); Clancy et al. (2020, 2023)^a^; Clancy, Klettke, et al. (2021)^a^; Clancy, Maas, et al. (2021); Gámez-Guadix et al. (2022); Hu et al. (2023); Kokkinos et al. (2024); Lu et al. (2021); Maes et al. (2023)^a^; Morelli et al. (2020, 2021, 2024); Seto et al. (2023); Wachs et al. (2021)
Nonsignificant	5	Califano et al. (2022); Clancy et al. (2020)^a^; Clancy, Klettke, et al. (2021)^a^; Ojeda et al. (2019); Said and McNealy (2023)
Negative association	1	Maes et al. (2023) ^a^
Receiving sexts		
Positive association	6	Badenes-Ribera et al. (2024); Casas et al. (2019); Clancy et al. (2020, 2023)^a^; Clancy, Klettke, et al. (2021)^a^; Clancy, Maas, et al. (2021); Seto et al. (2023)
Nonsignificant	3	Clancy et al. (2020)^a^; Clancy, Klettke, et al. (2021)^a^; Ojeda et al. (2019)
Pressured sexting		
Positive association	6	Kokkinos et al. (2024); Lu et al. (2021); Morelli et al. (2020, 2021, 2024); Wachs et al. (2021)
Receiving unwanted sexts		
Positive association	5	Boer et al. (2021); Clancy et al. (2023); Clancy, Klettke, et al. (2021); Lu et al. (2021); Said and McNealy (2023)
Risky sexting		
Positive association	3	Morelli et al. (2020, 2021, 2024)
Nonsignificant	1	Said and McNealy (2023)
Requesting sexts		
Positive association	6	Badenes-Ribera et al. (2024); Clancy et al. (2020, 2023)^a^; Clancy, Klettke, et al. (2021); Clancy, Maas, et al. (2021); Hu et al. (2023); Lu et al. (2021)
Nonsignificant	1	Clancy et al. (2023) ^a^
Aggression, victimization, and risk-taking		
Bullying perpetration		
Positive association	3	Gámez-Guadix et al. (2022); Hu et al. (2023); Ojeda et al. (2019)^a^
Negative association	1	Clancy, Maas, et al. (2021)
Nonsignificant	1	Ojeda et al. (2019) ^a^
Bullying victimization		
Positive association	1	Gámez-Guadix et al. (2022)
Negative association	1	Clancy, Maas, et al. (2021)
Nonsignificant	1	Hu et al. (2023)
Alcohol use		
Nonsignificant	3	Klein and Cooper (2019); Silva et al. (2020); Sparks et al. (2023)
Drug use		
Positive association	2	Silva et al. (2020); Sparks et al. (2023)
Impulsivity		
Positive association	2	Lu et al. (2021); Sparks et al. (2023)
(Low) self-control		
Nonsignificant	2	Holt et al. (2021); Morelli et al. (2024)
Emotional victimization		
Positive association	2	Barroso et al. (2021); Marinho et al. (2023)
Physical victimization		
Positive association	2	Barroso et al. (2021); Marinho et al. (2023)
Sexual victimization		
Positive association	2	Barroso et al. (2021); Marinho et al. (2023)
Anger and hostility		
Positive association	2	Barroso et al. (2021); Lu et al. (2021)
Aggressiveness		
Positive association	2	Barroso et al. (2021); Lu et al. (2021)
Peer problems		
Positive association	1	Barroso et al. (2021)
Nonsignificant	1	Badenes-Ribera et al. (2024)
Prosocial behavior		
Negative association	1	Casas et al. (2019)
Nonsignificant	1	Badenes-Ribera et al. (2024)
Attitudes and beliefs		
NCSII offense-supportive cognitions		
Positive association	8	Clancy et al. (2019, 2023)^a^; Clancy, Klettke, et al. (2021); Karasavva and Forth (2022)^a^; Maes et al. (2023); Schokkenbroek et al. (2023)^a^; Seto et al. (2023); Sparks et al. (2023)
Nonsignificant	4	Clancy et al. (2019, 2023)^a^; Karasavva and Forth (2022)^a^; Schokkenbroek et al. (2023)^a^
Sexism		
Positive association	3	Agueli et al. (2024); Martínez-Bacaicoa et al. (2024); Morelli et al. (2016)
Moral disengagement		
Positive association	2	Califano et al. (2022); Ojeda et al. (2024)
Personality traits		
Narcissism		
Positive association	6	Clancy et al. (2019, 2020)^a^; Karasavva and Forth (2022); Maes et al. (2023)^a^; Morelli et al. (2021)^a^; Sparks et al. (2023)
Nonsignificant	3	Clancy et al. (2019)^a^; Maes et al. (2023)^a^; Morelli et al. (2021)^a^
Machiavellianism		
Positive association	4	Clancy et al. (2019, 2020)^a^; Morelli et al. (2021); Sparks et al. (2023)
Nonsignificant	3	Clancy et al. (2019, 2020)^a^; ; Karasavva and Forth (2022)
Psychopathy		
Positive association	5	Clancy et al. (2019, 2020)^a^; Karasavva and Forth (2022)^a^; Morelli et al. (2021); Sparks et al. (2023)
Nonsignificant	3	Clancy et al. (2019, 2020)^a^; Karasavva and Forth (2022)^a^
Empathy		
Negative association	4	Longobardi et al. (2025)^a^; Maes et al. (2023); Morelli et al. (2024)^a^; Silva et al. (2020)^a^
Nonsignificant	3	Longobardi et al. (2025)^a^; Morelli et al. (2024)^a^; Silva et al. (2020)^a^
Callousness		
Positive association	2	Barroso et al. (2021); Marinho et al. (2023)
Uncaring		
Negative association	2	Barroso et al. (2021); Marinho et al. (2023)
Sadism		
Positive association	1	Karassava and Forth (2022)
Nonsignificant	1	Sparks et al. (2023)
Agreeableness		
Negative association	1	Kokkinos et al. (2024)
Nonsignificant	1	Morelli et al. (2020)
Conscientiousness		
Negative association	2	Kokkinos et al. (2024); Morelli et al. (2020)
Openness to experience		
Negative association	2	Kokkinos et al. (2024); Morelli et al. (2020)
Extraversion		
Nonsignificant	2	Kokkinos et al. (2024); Morelli et al. (2020)
Psychological wellbeing		
Symptoms of depression		
Positive association	4	Barroso et al. (2021); Gámez-Guadix et al. (2022); Lu et al. (2021); Wachs et al. (2021)
Nonsignificant	2	Clancy et al. (2023); Clancy, Maas, et al. (2021)
Symptoms of anxiety		
Positive association	4	Barroso et al. (2021); Clancy et al. (2023)^a^; Clancy, Maas, et al. (2021); Gámez-Guadix et al. (2022)
Nonsignificant	2	Clancy et al. (2023)^a^; Lu et al. (2021)
Psychological distress		
Positive association	2	Clancy et al. (2023)^a^; Clancy, Maas, et al. (2021)
Nonsignificant	2	Clancy et al. (2023)^a^; Smith et al. (2018)
Negative association	1	Badenes-Ribera et al. (2024)
Self-esteem		
Nonsignificant	2	Sesar et al. (2021)^a^; Smith et al. (2018)
Negative association	2	Klein and Cooper (2019); Sesar et al. (2021)^a^
Body esteem		
Nonsignificant	2	Califano et al. (2022); Sesar et al. (2021)^a^
Positive association	1	Sesar et al. (2021) ^a^
General mental health		
Negative association	1	Boer et al. (2021)
Nonsignificant	1	Morelli et al. (2024)
Sexual behavior, porn, and dating app use		
(Lifetime) sexual activity		
Positive association	4	Boer et al. (2021); Clancy et al. (2019, 2020)^a^; Clancy, Maas, et al. (2021)
Nonsignificant	2	Clancy et al. (2020, 2023)^a^
Sexual debut age		
Negative association	2	Clancy et al. (2019, 2020)
Dating app use		
Positive association	3	Blais et al. (2025); Boer et al. (2021); Seto et al. (2023)^a^
Nonsignificant	1	Seto et al. (2023) ^a^
Pornography use		
Positive association	2	Boer et al. (2021); Holt et al. (2021)
Negative association	1	Maes et al. (2023)
Nonsignificant	1	Silva et al. (2020)

*Note.* Results are organized into general domains and listed according to frequency. Findings reported by Clancy, Klettke, et al. (2021) and Clancy, Maas, et al. (2021) are counted only once, as their analyses were based on the same dataset. However, both references are listed.

a Results were mixed across different levels of analysis, subgroups of participants, types of NCSII, and/or dimensions of the same construct.

b Results could not be synthesized due to considerable variations in comparison groups across studies.

NCSII = nonconsensual sharing of intimate images.

##### Sociodemographic Characteristics (*n* = 48)

Gender (*n* = 48) was the most widely studied covariate of NCSII perpetration. Over half of the studies found boys and men more likely to perpetrate NCSII than girls and women, while the rest reported no significant gender difference. Also, one study noted that boys were more likely to share intimate images if they had taken the images themselves but not if they had simply received them (Strassberg et al., 2013). Notably, none of the studies found that perpetration rates were highest among girls and women. For age (*n* = 33), roughly two-thirds of the studies reported nonsignificant findings, while the rest found positive and/or negative associations, two of which indicated a curvilinear relationship between age and NCSII perpetration (Patchin & Hinduja, 2019; Van den Eynde et al., 2023). Regarding sexual orientation (*n* = 21), two-thirds of the studies found no significant difference, while the rest reported higher rates among non-heterosexual than heterosexual participants. A handful of studies also explored associations with other sociodemographic factors, including ethnicity/race (*n* = 10), relationship status (*n* = 5), nationality (*n* = 4), level of education (*n* = 4), area of residence (*n* = 3), and socioeconomic status (*n* = 2), all of which yielded either nonsignificant or mixed findings.

##### Intimate Image-Sharing Experiences (*n* = 28)

The most robust finding across studies was that perpetrators were more likely than non-perpetrators to be victims (*n* = 14) and bystanders (*n* = 9) of NCSII. There was also a longitudinal association with previous NCSII perpetration (*n* = 3). Moreover, for sending (*n* = 19), receiving (*n* = 7), and requesting (*n* = 6) sexts, most studies reported positive associations, though some reported that findings were nonsignificant or inconsistent across different analyses or subgroups of participants (e.g., Clancy, Klettke, et al., 2021). Results for pressured (*n* = 6), unwanted (*n* = 5), and risky sexting (i.e., sending sexts to strangers or while intoxicated; *n* = 4) were fewer but more consistent, as nearly all studies reported positive associations, with the exception of risky sexting in Said and McNealy’s (2023) study.

##### Aggression, Victimization, and Risk Behavior (*n* = 14)

While numerous studies examined the relationship between NCSII perpetration and various forms of aggression, victimization, and risk behavior, findings from this line of research were considerably fragmented and inconsistent. With regard to bullying perpetration (*n* = 4) and victimization (*n* = 3), peer problems (*n* = 2), and prosocial behavior (*n* = 2), results varied both within and across studies. However, positive associations were invariably found for drug use (*n* = 2), impulsivity (*n* = 2), emotional, physical, and sexual victimization (*n* = 2), as well as anger, hostility, and aggression (*n* = 2). Associations with alcohol use (*n* = 3) and self-control (*n* = 2) were, on the other hand, consistently found to be nonsignificant.

##### Social Cognitive Factors (*n* = 13)

In the last five years, several studies have explored the relationship between NCSII perpetration and various offense-supportive cognitions (e.g., attitudes and beliefs) related to intimate image sharing practices (*n* = 8). However, findings were inconsistent, indicating a mix of positive and nonsignificant associations both across and within studies (e.g., Clancy et al. 2023), possibly because of variations in how these cognitions have been operationalized (e.g., as subjective norms, myth acceptance, or victim blame) and analyzed (e.g., item or scale level). Regarding other social cognitive factors, studies were scarce but consistently reported positive associations with sexism (*n* = 3) and moral disengagement (*n* = 2).

##### Personality Traits (*n* = 13)

With regard to personality, the overall findings indicated positive associations between NCSII perpetration and narcissism (*n* = 6), psychopathy (*n* = 5), Machiavellianism (*n* = 5), sadism (*n* = 2), and callousness (*n* = 2), and negative associations with empathy (*n* = 4), conscientiousness (*n* = 2), agreeableness (*n* = 2), and openness to experience (*n* = 2). However, it should be noted that for the majority of these covariates, results varied across different studies, facets of the same construct, and levels of analyses, with multiple regression models frequently yielding nonsignificant associations (e.g., Maes et al., 2023). Also, studies that examined associations with extraversion (*n* = 2) indicated that this personality trait was not connected to NCSII perpetration.

##### Psychological Well-Being (*n* = 13)

Concerning mental health, a handful of studies examined the relationship between NCSII perpetration and symptoms of depression (*n* = 6) and anxiety (*n* = 5), psychological distress (*n* = 4), self-esteem (*n* = 3), body esteem (*n* = 2), and general mental health (*n* = 2). Again, results for all indicators were considerably inconsistent, both within and across studies, making it challenging to synthesize findings.

##### Sexual Behavior, Porn, and Dating App Use (*n* = 10)

Few studies explored the relationship with lifetime sexual activity (*n* = 5), sexual debut age (*n* = 2), pornography (*n* = 4), or dating app (*n* = 3) use. Based on bivariate analyses, all studies found that NCSII perpetrators were more likely to be sexually active and debut earlier than non-perpetrators. However, when Clancy et al. (2020) included sexual activity in a multiple regression model, the association was no longer significant. Likewise, all studies that explored dating app use found a positive association, though results from a multiple regression analysis by Seto et al. (2023) indicated that the relationship was no longer significant when controlling for other variables. With regard to pornography use, findings were markedly inconsistent, with results showing negative, positive, and nonsignificant associations.

#### Perpetrator Motives

In total, six studies explored perpetrators’ motives for engaging in NCSII (Barrense-Dias et al., 2020; Clancy et al., 2019, 2020, 2023; Clancy, Klettke, et al., 2021; Walker et al., 2021). Across studies, the most widely endorsed motives were related to amusement (22%–62%), carelessness (2%–48%), showing off (5%–44%), social belonging (1%–23%), social support (4%–19%), social rewards (5%–16%), and roasting or teasing (9%–14%). Overall, very few perpetrators reported sharing intimate images of others for sexual reasons (2%–7%) or out of malice, spite, or revenge (1%–7%). Overall, gender comparative analyses showed that a significantly higher proportion of boys and men indicated that they had wanted to “show off”, compared to girls and women (e.g., Barrense-Dias et al., 2020). Interestingly, the inverse was frequently found for motives connected to roasting or teasing, and malice, spite, or revenge, which were primarily endorsed by female perpetrators, though gender differences were not consistently significant (e.g., Clancy et al., 2021, 2023).

### Qualitative Findings

In total, we identified 13 qualitative studies that reported analyses of statements by, and/or interactions between, NCSII perpetrators. Summary tables of the key characteristics, findings, and quality assessments of all studies can be found in the Supplemental Materials. In short, five of the studies used digital ethnography, three used semi-structured interviews, and five combined one or both of these methods with fieldwork or focus group interviews. All but one of the studies (Ringrose et al., 2013) were published between 2019 and 2024. While not all studies involved participants, those that did collected data from a total of 455 individuals (boys/men = 233, girls/women = 186, non-binary = 1, unknown = 35) between the ages of 12 and 25 years. The studies were conducted in the United Kingdom (*n* = 3), Denmark (*n* = 2), the Netherlands (*n* = 1), Sweden (*n* = 1), and the United States (*n* = 1), as well as on various online platforms (*n* = 5). Quality issues primarily concerned a lack of transparency about the analysis process, and incoherences in the studies’ theoretical framework, research methods, and interpretations.

#### Social Contexts and Dynamics

Table 2 lists the themes developed through our qualitative synthesis (*n* = 13) and shows how they were represented across the included studies. To maintain brevity, the below summary is primarily structured around the main themes, with the exception of “A Manhood Act,” which was the most prominent theme and therefore warrants a more detailed synopsis.

**Table 2.** Themes Identified Through Thematic Synthesis.

Main theme	Subtheme	References												
		Hall and Hearn (2019)	Hall et al. (2022)	Harder (2021)	Hearn and Hall (2019)	Hunehäll Berndtsson (2021)	Johnstonbaugh (2021)	Naezer and van Oosterhout (2021)	O’Rourke and Haslop (2025)	Otteren and Gynnhild (2021)	Rasmussen and Søndergaard (2021)	Ringrose et al. (2013)	Semenzin and Bainotti (2020)	Storrod (2024)
A manhood act	Girls’ and women’s bodies as valuable items		x	x		x	x	x	x	X	x	x	x	x
	Heteronormative camaraderie and competition	x	x	x	x		x	x	x	X	x	x	x	
	Male dominance and misogyny	x			x		x	x	x	X	x	x	x	x
A targeted attack	Nudes as loaded weapons					x		x						
	Aggression, contempt, and moralism	x			x	x		x						
A lack of accountability	Victim blaming	x	x	x				x					x	
	Ignorance, immaturity, and impulsivity								x				x	
	Digital affordances									X			x	
A coping strategy	Cyberflashing and sext requests						x	x						
	Support through communal ridicule and disgust			x			x	x						

*Note.* NCSII = nonconsensual sharing of intimate images.

##### A Manhood Act (*n* = 13)

The primary theme of our synthesis draws on research showing that boys and men share intimate images of girls and women to assert and reinforce their masculinity, and thus position themselves within gendered social hierarchies.

##### Girls’ and Women’s Bodies as Valuable Items

A common thread across studies (*n* = 11) was male perpetrators’ collective treatment of the female body as a prized possession that they could freely ogle, seize, collect, and trade. For example, several studies found that boys and men readily commodified female nudes by exchanging them with peers as a form of trading cards or social currency (e.g., O’Rourke & Haslop, 2025). This tendency indicates that male entitlement and female sexual objectification may be central drivers of NCSII incidents.

Paradoxically, studies exploring user interactions on online forums found that images that had been purposefully created by women to capitalize on their own sexuality were attributed far less value than NCSII content. Rasmussen and Søndergaard (2021) suggest that the desirability of the latter may be tied to the perceived authenticity and limited accessibility of private intimate imagery. Relatedly, focus group and interview research indicated that receiving nudes from “ordinary” girls and women was perceived as an enviable achievement among boys and men, representing a form of personal validation (e.g., Johnstonbaugh, 2021).

##### Heteronormative Camaraderie and Competition

The included studies also indicated that male perpetrators may reap social rewards by sharing intimate images of girls and women (*n* = 11). One key incentive appears to be homosocial bonding, as studies found that NCSII was intentionally perpetrated to show loyalty, foster platonic intimacy, and gain reverence from other boys and men (e.g., Naezer & Van Oosterhout, 2021). An extreme version of this dynamic was observed on online platforms, where NCSII incidents manifested as “scavenger hunts” in which multiple perpetrators collaborated to obtain female nudes of named victims (e.g., Otteren, & Gynnhild, 2021). Notably, user interactions on these forums were found to be characterized by references to masculinity ideals (e.g., bravery; Hall et al., 2022) and military terminology (Semenzin & Bainotti, 2020), potentially fostering a sense of male solidarity.

Furthermore, several studies found that male perpetrators treated intimate images of girls and women as “trophies” that could be shown off to signal their success *to* and *over* other boys and men (e.g., Johnstonbaugh, 2021). Across studies, researchers tended to link this practice to contemporary ideals of heteronormative masculinity, in which sexual pursuit and conquest are considered to be central elements (e.g., O’Rourke & Haslop, 2025). Consonant with this, male perpetrators were found to receive praise, admiration, and signs of respect from their same-sex friends upon sharing the images with them (e.g., Harder, 2021)

While much rarer than the above patterns, two studies reported examples of female perpetrators engaging in NCSII in the pursuit of peer validation. In line with the above tendencies, Naezer and van Oosterhout (2021) found that a girl in their study had tried to capitalize on the social currency of female nudes by sharing images of a same-sex peer with a boy she knew, hoping to gain acceptance from him and his friends. By contrast, Johnstonbaugh (2021) found that some women shared intimate images of men to brag about their sexual conquests to their female friends. However, women rarely responded enthusiastically to this, indicating that male nudes do not hold the same value for women as female nudes do for men.

##### Male Dominance and Misogyny

Findings also pointed toward male dominance and misogyny as important social dynamics in male-perpetrated NCSII (*n* = 10). This was most evident in studies of online communities dedicated to sharing intimate imagery of girls and women (e.g., Otteren & Gynnhild, 2021). In this context, misogyny manifested in the use of derogatory terms, such as “bitch,” “slut,” “whore,” “cunt,” and “cum bucket,” which appeared to be standard vernacular on these websites and forums (e.g., Rasmussen & Søndergaard, 2021). Reflecting on this trend, Semenzin and Bainotti (2020, p. 7) concluded that NCSII in these networks represents “a group practice of denigration, humiliation, and derision in a male homosocial environment.” Assertions of misogyny were also documented in research on revenge pornography sites (e.g., Hall & Hearn, 2019) and, to a lesser extent, in focus group studies (e.g., Storrod, 2024).

Interestingly, some studies indicated that boys and men would treat female nudes differently depending on the nature of their relationship with the person in the image (e.g., Naezer & van Oosterhout, 2021). More specifically, it was reported that current girlfriends were less likely to be subjected to NCSII compared to strangers, acquaintances, exes, and friends (e.g., O’Rourke & Haslop, 2025). This tendency seems to imply that some male perpetrators of NCSII do not view girls’ and women’s right to autonomy, integrity, and privacy as inherent, but rather as something that is granted through their emotional connection with them and other boys and men.

##### A Targeted Attack (*n* = 4)

Based on data richness, the second largest theme of our synthesis concerns the deliberate weaponization of intimate imagery to harm other people’s reputation. Importantly, studies found that, in this context, NCSII was not necessarily carried out in response to some perceived offense, suggesting that these perpetrators are not exclusively motivated by “revenge.” For instance, Hunehall Berndtsson’s (2021) study showed that NCSII can transpire as a form of bullying, where adolescents target their peers simply because they dislike them. Studies also found that NCSII can serve as a tool for social regulation and punishment. In particular, girls were found to use NCSII as a way of policing behaviors they perceived as reckless, inappropriate, or immoral (e.g., Naezer & Van Oosterhout, 2021).

On the other hand, studies of so-called “revenge pornography” websites found that the texts published alongside intimate photos and videos were, overall, laden with expressions of aggrievement. Typically, perpetrators presented themselves as the scorned ex-partners of their victims, whom they characterized as cruel, worthless, or immoral (e.g., Hall & Hearn, 2019). Studies also included numerous examples of perpetrators shaming victims for their sexual skills, appetite, or preferences—often in highly gendered ways: whereas female victims were vilified for being inexperienced, frigid, or promiscuous, male victims were mocked for being “kinky” and not conforming to normative heterosexual practices (e.g., Hearn & Hall, 2019).

##### A Lack of Accountability (*n* = 7)

The third main theme of our synthesis concerns conditions that may diminish perpetrators’ sense of accountability, thus potentially lowering the threshold for engaging in NCSII. Most notably, findings indicated that perpetrators may shift responsibility and blame for the incident onto the victim’s actions, possibly to minimize the severity of their own transgression (e.g., Harder, 2021). In addition to this, some studies pointed to ignorance, immaturity, and impulsivity as factors that may reduce perpetrators’ sense of responsibility for NCII incidents (e.g., O’Rourke & Haslop, 2025). Lastly, findings from two studies of online communities suggested that the anonymity provided by online spaces functions as a “protection mechanism” that fosters a sense of impunity among users (Otteren & Gynnhild, 2021; Semenzin & Bainotti, 2020).

##### A Coping Strategy (*n* = 3)

The final theme of our synthesis concerns a form of NCSII that only a handful of studies addressed, namely, sharing intimate imagery as a way to cope with image-based sexual harassment. This small body of research primarily concerned situations in which a girl or woman had received unwanted sexual photos or videos (e.g., dick pics) from a male friend, acquaintance, or stranger, which was typically described by them as “shocking,” “disrespectful,” “uncomfortable,” “weird,” “gross,” and “dirty” (e.g., Naezer & van Oosterhout, 2021). In response, some girls and women had shared and ridiculed the unsolicited images with their close friends. In one sense, this form of NCSII can be seen as an act of revenge aimed at humiliating the boy or man who sent the image. However, these studies also indicate that such actions are primarily motivated by a need to regain a sense of control after being subjected to a distressing violation (e.g., Johnstonbaugh, 2021).

## Discussion

This review aimed to synthesize existing research on the psychosocial mechanisms of NCSII, focusing on empirical findings regarding the correlates, motives, and social dynamics of perpetration. In the results section, we reviewed findings from qualitative and quantitative studies separately, exploring the nuances within each body of knowledge. In the following, we distill, connect, and contrast key insights from each strand of the synthesis to further theorize around the driving forces of NCSII perpetration and identify critical gaps in, and future directions for, research on this topic. For summaries of critical findings and implications, see Tables 3 and 4.

**Table 3.** Critical Findings.

Domain	Summary
Perpetrator characteristics	• Research on potential covariates spans multiple domains, including sociodemographic characteristics; intimate image-sharing practices; aggression, victimization, and risk behavior; social cognitive factors; personality traits; psychological well-being; and sexual behavior, porn, and dating app use, but results are fragmented and inconsistent • The most robust finding is that there is an overlap between NCSII perpetration and victimization, as well as exposure to and experience with other intimate image-sharing practices • While gender is not a reliable predictor, none of the studies found that girls are more likely than boys to perpetrate NCSII
Perpetrator motives	• Reasons typically relate to amusement, carelessness, and showing off • Male perpetrators are more likely than female perpetrators to report motives related to showing off, while the opposite was found for motives related to roasting or teasing, and malice, spite, or revenge
Social context and dynamics	• Qualitative studies indicate that male-perpetrated NCSII transpires as a form of homosocial bonding, situated in hegemonic masculinity, male entitlement, female sexual objectification, and sexism • Ill-intentioned acts of NCSII are not only driven by revenge but also transpire as a form of bullying and social control • Girls and women may perpetrate NCSII against boys and men as a way to cope with image-based sexual harassment

*Note.* NCSII = nonconsensual sharing of intimate images.

**Table 4.** Implications for Research and Practice.

Domain	Summary
Policy	• Laws criminalizing NCSII should not focus exclusively on actions driven by ill intentions or revenge
Practice	• Efforts to prevent NCSII should address the social dynamics between perpetrators and bystanders, as well as issues pertaining to the broader cultural context of these interactions, including hegemonic masculinity, male entitlement, female sexual objectification, and sexism • Preventive measures should also focus on reducing image-based sexual harassment and providing potential victims with information about how to deal with such transgressions in a way that does not involve additional violations
Research	• The field would benefit from further quantitative research on social cognitions and gender differences in covariates of NCSII perpetration • To better understand gender differences in the social dynamics of NCSII, we need more qualitative research on female perpetrators • The time is ripe for more focused reviews on NCSII perpetration, including meta-analyses that examine specific associations

*Note.* NCSII = nonconsensual sharing of intimate images.

### Driving Forces of NCSII Perpetration

Our synthesis of quantitative studies revealed that current research on the characteristics of NCSII perpetrators is fragmented and inconsistent. These issues likely stem from both the novelty of the topic and variations in how NCSII and its covariates have been operationalized across studies. There were, however, a few notable exceptions. In particular, we found that all analyses of the relationship between NCSII perpetration and victimization showed a positive association (e.g., Boer et al., 2021), making it the most robust finding across studies. The concurrence of these experiences—commonly referred to as the victim-offender overlap—is also widely documented in research on other forms of interpersonal violence, with various models put forth to explain the phenomenon (Jennings et al., 2012).

According to routine activity theory and subcultural theories of delinquency, individuals who are embedded in environments with higher exposure to, and norms that condone, transgressive behaviors are more likely to be both victims and perpetrators (Berg & Mulford, 2020). Echoing this, studies included in this review consistently found that exposure to and experiences with various intimate image-sharing practices were more common among perpetrators than non-perpetrators (e.g., Clancy et al., 2023), and that some of the most frequently endorsed motives for NCSII were related to amusement, showing off, social belonging, and social rewards (e.g., Barrense-Dias et al., 2020). The latter indicates that perpetrators may view NCSII as socially acceptable, which resonates with findings from the included qualitative studies showing that perpetrators may enjoy a range of social rewards in response to sharing intimate images of others (e.g., Harder, 2021).

Taken together, the above findings indicate that contextualized approaches may be particularly useful for efforts to understand and address NCSII perpetration. Notably, the importance of context was widely recognized by the included qualitative studies, as many took into consideration the cultural backdrop and social dynamics of NCSII incidents, highlighting the influence of hegemonic masculinity, sexism, and sexual objectification (e.g., Ringrose, 2013). However, the findings of these studies do not answer the question of why some, but not all, individuals in the same setting choose to share intimate images of others without their consent. Hence, to better understand mechanisms of perpetration, the field may benefit from research examining individual differences in social cognitive factors that, theoretically, could lead to the normalization and trivialization of these incidents.

Within the current literature, we identified only a handful of quantitative studies that explored how such cognitions were connected to NCSII perpetration. While results regarding sexism (e.g., Agueli et al., 2024) and moral disengagement (e.g., Ojeda et al., 2024) all showed positive associations, findings were mixed for offense-supportive cognitions specific to NCSII incidents (e.g., Karasavva & Forth, 2022). As briefly noted in the results section, one reason for this may be that none of the included studies used the same instrument to measure these attitudes and beliefs. Another issue pertains to the development and validity of these measures, which were barely addressed by the included studies. To better understand how offense-supportive cognitions are associated with NCSII perpetration, further efforts should be made to systematically develop and assess the validity of psychometric instruments.

It is also noteworthy that quantitative studies consistently found that only a fraction of perpetrators reported that they had wanted to harm or humiliate their victims (e.g., Clancy, Klettke et al., 2021). This might help explain why results concerning the unique predictive power of dark personality traits have been inconsistent. It also indicates that purely psychological theories developed to explain socially deviant behaviors may not be the most useful frameworks for understanding NCSII perpetration. Instead, our synthesis points toward gender roles and homosocial bonding as highly relevant theoretical constructs. Indeed, although sex and gender do not appear to be robust predictors of perpetration, both quantitative and qualitative studies show that gender plays a crucial role in the motives and social dynamics of NCSII incidents, indicating that the act of sharing intimate images of others without their consent holds gendered meanings.

Specifically, quantitative findings suggest that boys and men are more likely than girls and women to engage in NCSII perpetration as a way to show off and bond with their male friends (e.g., Clancy, Klettke et al., 2021), while qualitative findings illustrate how these dynamics may be shaped by and reproduce social dynamics grounded in hegemonic masculinity and female sexual objectification (e.g., Semenzin & Bainotti, 2020). On the other hand, the overall findings indicate that girls and women are more likely than boys and men to engage in NCSII to police the behaviors of their peers or seek social support after experiencing image-based sexual harassment (e.g., Naezer & Van Oosterhout, 2021). It is also worth noting that quantitative studies found a connection between NCSII perpetration and sexism (e.g., Agueli et al., 2024), further underscoring the relevance of gender belief systems. While more research is needed to better understand these mechanisms, the findings of this review highlight the importance of adopting a gender-sensitive approach in NCSII prevention.

### Strengths and Limitations

The present study offers the most comprehensive review of research on NCSII perpetration to date. While our findings resonate with Henry and Beard’s (2024) recent review of research concerning image-based sexual abuse more broadly, they also extend beyond these by further elucidating the interconnectedness between NCSII perpetration and other intimate image-sharing practices, as well as social cognitions and dynamics that foster normalization and trivialization of NCSII incidents. Nevertheless, the following limitations should be taken into consideration when evaluating the contributions of this review.

Regarding the quantitative synthesis, it should be noted that the use of vote-counting has some considerable shortcomings, in that it only considers the direction and significance of associations (Hedges, 1980). Hence, the field would benefit from additional reviews on the characteristics of NCSII perpetrators that employ meta-analytical methods. As for the qualitative strand, we want to acknowledge that the use of thematic synthesis takes for granted a shared reality and transferability of findings across primary studies, irrespective of methodological differences, and therefore does not take into consideration interpretive and constructionist aspects of the knowledge production process (Barnett-Page & Thomas, 2009). This is particularly pertinent to consider because our analysis was not only based on data extracts from NCSII perpetrators, but also on excerpts of researchers’ own interpretations. As such, the findings of the qualitative synthesis are inevitably influenced by the theoretical perspectives adopted in the included studies.

For the review as a whole, key limitations include the exclusive focus on English-language articles published in peer-reviewed journals, which may have resulted in relevant research (e.g., gray literature) not being included in the review and thus introduced cultural bias into our analysis. It is also important to note that while the review was inclusive with regard to the race, ethnicity, nationality, and gender identity (etc.) of primary research participants, these characteristics receive little attention as very few of the included studies addressed them, and those that did focused on different subgroups and/or produced mixed findings. With regard to methodology, it is noteworthy that around a third of the articles were screened by one reviewer only, which may have increased the risk of bias. However, this approach was deemed necessary, as we did not have sufficient resources to double-screen records from the final literature search.

Lastly, while the results section presents several noteworthy findings, these are only partially unpacked in the discussion. This limitation reflects our intentionally broad research questions, which were devised to identify the psychosocial mechanisms of NCSII perpetration most consistently supported by current evidence. Consequently, a detailed investigation of the underlying reasons for inconsistent results across quantitative studies fell outside the scope of this review. As previously noted, the empirical literature is highly fragmented—both with regard to terminology and operationalizations—thus complicating cross-study comparisons. To further evaluate the robustness of the identified covariates of NCSII perpetration, the field may benefit from even more focused reviews going forward.

## Conclusion

By juxtaposing quantitative and qualitative research on NCSII perpetration, the present review offers a comprehensive synthesis of current empirical insights concerning its underlying mechanisms. In sum, our findings suggest that NCSII perpetration is associated with other intimate image-sharing experiences (e.g., NCSII victimization), and primarily transpires as a form of homosocial bonding that is situated within a wider social context of hegemonic masculinity, female sexual objectification, and sexism. Hence, efforts to study and address NCSII would likely benefit from taking into consideration the social and cultural context of perpetrators’ actions, including gender roles and dynamics, as well as normative beliefs about and experiences with various intimate image-sharing practices.

## Supplemental Material

sj-docx-1-tva-10.1177_15248380251375912 – Supplemental material for Identifying Drivers of Nonconsensual Intimate Image Sharing: A Mixed-Methods Synthesis of Perpetrator Research Supplemental material, sj-docx-1-tva-10.1177_15248380251375912 for Identifying Drivers of Nonconsensual Intimate Image Sharing: A Mixed-Methods Synthesis of Perpetrator Research by Stine Nygård, Maren Kristin Nysæter, Ligia Ribeiro Ferreira, Lars Roar Frøyland and Ingela Lundin Kvalem in Trauma, Violence, & Abuse
