# Supplementary material for: Identifying Drivers of Nonconsensual Intimate Image Sharing: A Mixed-Methods Synthesis of Perpetrator Research
Source: Trauma Violence Abuse. 2025 Oct 4;27(5):2161–79. doi: 10.1177/15248380251375912 (PMC13736305; doi:10.1177/15248380251375912)
Supplement: Supplementary file 1 [file sj-docx-1-tva-10.1177_15248380251375912.docx]

**Supplemental Materials for**

**“Identifying Drivers of Nonconsensual Intimate Image Sharing:
A Mixed Methods Synthesis of Perpetrator Research”**

Table of Contents

[Appendix A Labels for motives reported by NCSII perpetrators	2](#_Toc188088621)

[Table A1 Connection between labels used for motives in the synthesis and primary studies	2](#_Toc188088622)

[Appendix B Example of analytical steps in thematic synthesis	3](#_Toc188088623)

[Appendix C Characteristics and Quality Assessment of Quantitative Studies	4](#_Toc188088624)

[Table C1 Key characteristics and findings of quantitative studies (n = 55)	4](#_Toc188088625)

[Table C2 Summary of quality assessment of quantitative studies (n = 55)	19](#_Toc188088626)

[Appendix D Characteristics and Quality Assessment of Quantitative Studies	21](#_Toc188088627)

[Table D1 Key characteristics and findings of qualitative studies (n = 13)	21](#_Toc188088628)

[Table D2 Summary of quality assessment of qualitative studies (n = 13)	26](#_Toc188088629)

# Appendix A Labels for motives reported by NCSII perpetrators

## Table A1 *Connection between labels used for motives in the synthesis and primary studies*

| Categories of motives | Reported reasons and motives | Reference(s) |
| --- | --- | --- |
| Amusement | As a joke, to be funny | Clancy et al., (2019, 2020, 2023); Clancy, Klettke, et al. (2021) |
|  | For fun | Barrense-Dias et al. (2020) |
|  | For fun/as a joke | Walker et al. (2021) |
| Carelessness | Did not think it was a big deal | Clancy et al. (2019, 2020, 2023); Clancy, Klettke, et al. (2021) |
|  | Not realizing what they were doing | Barrense-Dias et al. (2020) |
|  | Shared in error | Clancy et al. (2020) |
| Showing off | Because the person was hot | Clancy et al. (2020; 2023); Clancy, Klettke, et al. (2021) |
|  | To brag | Clancy et al. (2020; 2023); Clancy, Klettke, et al. (2021) |
|  | To show off | Barrense-Dias et al. (2020); Walker et al. (2021) |
|  | To prove that they received it | Clancy et al. (2023); Walker et al. (2021) |
| Social belonging | Because of pressure to share | Barrense-Dias et al. (2020); Clancy et al. (2019, 2020; 2023); Clancy, Klettke, et al. (2021) |
|  | Because they were asked to share | Clancy et al. (2019, 2020, 2023); Clancy, Klettke ,et al. (2021); Walker et al. (2021) |
|  | To gossip | Clancy et al. (2020, 2023); Clancy, Klettke, et al. (2021) |
|  | Because they always share things like this with friends | Walker et al. (2021) |
|  | Being accepted in a group | Barrense-Dias et al. (2020) |
| Social support | Asking for friends’ opinion | Barrense-Dias et al. (2020) |
|  | To demonstrate harassment | Clancy et al. (2020) |
|  | To ask for advice, seek an opinion, or discuss | Walker et al. (2021) |
| Social rewards | To improve social status | Clancy et al. (2019, 2020, 2023); Clancy, Klettke, et al. (2021); |
|  | To get attention/praise | Clancy et al. (2019, 2020, 2023); Clancy, Klettke, et al. (2021); |
|  | To get attention | Walker et al. (2021) |
| Roasting or teasing | To roast or tease the person depicted | Clancy et al. (2023); Clancy, Klettke, et al. (2021) |
|  | Roast | Clancy et al. (2020) |
| Malice, spite, or revenge | To get the recipient in trouble | Clancy et al. (2019, 2020, 2023); Clancy, Klettke, et al. (2021) |
|  | To get back at the person/get revenge | Clancy et al. (2020, 2023); Clancy, Klettke, et al. (2021) |
|  | Out of spite | Clancy et al. (2020, 2023); Clancy, Klettke, et al. (2021) |
|  | Jealousy/meanness | Barrense-Dias et al. (2020) |
|  | Love revenge | Barrense-Dias et al. (2020) |
| Sexual reasons | To initiate sexual contact | Clancy et al. (2020, 2023); Clancy, Klettke, et al. (2021) |
|  | To “spice up” relationship | Clancy et al. (2020) |

# Appendix B Example of analytical steps in thematic synthesis

The analytical steps of thematic synthesis involves (1) coding data extracts inductively and generating (2) descriptive and (3) analytical themes (Thomas & Harden, 2008). This process is exemplified below, using a quote from Harder’s study (2021, p. 655):

Data extract:	“You know, it’s like you are with your boys and they are like ‘sure, okay’, and ‘yeah, great’ and ‘can I see it?’ and that kinda thing. And then – you know, you want to please them (…)”

Initial code:	Complying with male friends

Descriptive theme:	Masculine camaraderie and competition

Analytical theme:	(NCSII as) a manhood act

# Appendix C Characteristics and Quality Assessment of Quantitative Studies

## Table C1 *Key characteristics and findings of quantitative studies (*n *= 55)*

|  |  |  |  |  | Key findings | |
| --- | --- | --- | --- | --- | --- | --- |
| Author(s) | Design | Country | Sample | Measure of NCSII | Bivariate analyses | Multiple regression |
| Agueli et al. (2024) | Cross-sectional | Italy | 2,305 adults  Age = 18-35 years  Female = 55% Male = 45% | Six items (e.g., “Have you ever sent sexual or provocative photos/videos of your partner via messaging apps [SMS, WhatsApp, Telegram, etc.], without their permission”) from the Sexting Behaviours Questionnaire^a^. Items were responded to with a 5-point scale ranging from “never” to “frequently or daily”. | Sex/gender (male > female); sexism (+) |  |
| Badenes-Ribera et al. (2024) | Cross-sectional | Italy | 999 adults  Age = 18-35 years  Female = 76% Male = 20.7% Non-binary = 3.3% | One item (“Have you ever received an image-based sext that was intended for yourself that you subsequently showed/sent to another person?”)^b^. Response options were “yes” and “no”. | Sex/gender (male > female); age (+); sexual orientation (non-heterosexual > heterosexual); NCSII victimization (+); receiving NCSII (+); sending sexts (+); receiving sexts (+); requesting sexts (+);peer problems (ns); prosocial behavior (ns); psychological distress (-) |  |
| Barrense-Dias et al. (2020) | Cross-sectional | Switzerland | 5,175 adults  Age = 24-26 years  Female = 49% Male = 51% | One item (“Have you ever shared [forwarding or showing] a sexy photograph/ video of someone else [known or unknown] without consent?”). The term ‘sexy’ was deﬁned as “sexually suggestive, sexually implicit, nude, semi-nude, dressed, ﬂirtatious, etc.”. Response options were “never”, “once”, “several times”. | Sex/gender (male > female); age (ns); sexual orientation (non-heterosexual > heterosexual); ethnicity/race (foreign-born > not foreign-born); level of education (-); socioeconomic status (ns); area of residence (ns); receiving NCSII (+); sending sexts (+) |  |
| Barroso et al. (2021) | Cross-sectional | Portugal | 4,281 adolescents  Age = 12-20 years  Female = 52.9% Male = 47.1% | One item (“Have you ever shared [forwarding or showing] a sexy photograph/video of someone else [known or unknown] without consent?”). Response options were “yes” and “no”. | Sex/gender (male > female); age (+); socioeconomic status (ns); NCSII victimization (+); anger and hostility (+); aggressiveness (+); peer problems (+); emotional victimization (+); physical victimization (+); sexual victimization (+); callousness (+); uncaring (-); symptoms of anxiety and depression (+) |  |
| Bianchi et al. (2016) | Cross-sectional | Italy | 509 adolescents and adults who had sexted in the last year  Age = 13-35 years  Female = 63.7% Male = 36.3% | One item about sharing a partner’s sexts without consent from the Sexting Behaviours Questionnaire^a^. The exact wording of the item is not provided. The item was responded to with a 5-point scale ranging from “never” to “frequently or daily”. |  | Sex/gender (male > female); age (ns); sexual orientation (ns) |
| Blais et al. (2024) | Cross-sectional | Canada | 1,200 adults  Age = 18-84 years  Female = 47% Male = 50.6% Gender diverse = 2.3% | Four items (e.g., “Have you privately shared sexually suggestive or naked photos/videos of your partner without their consent”) from the Sexting Behaviours Questionnaire^a^. Limited to experiences in the 12 months prior to the survey. Response options were “yes” and “no”. | Sex/gender (male > female); age (ns); sexual orientation (ns); ethnicity/race (ns); relationship status (ns); dating app use (+) |  |
| Boer et al. (2021) | Cross-sectional | Netherlands | 20,834 adolescents and adults  Age = 12-24 years  Female = 60.7% Male = 39.3% | One item (asking if respondents “had ever sent a nude image or sex-video of someone else within the last six months”). Response options were “never”, “once”, “more than once”. Responses were dichotomized to “never” and “once/more than once” prior to analysis. |  | Sex/gender (male > female); age (-); sexual orientation (ns); ethnicity/race (non-western > western); level of education (-); area of residence (rural, urban-rural > urban); NCSII victimization (+); receiving unwanted sexts (+); general mental health (-); sexual activity (+); dating app use (+); pornography use (+) |
| Califano et al. (2022) | Cross-sectional | Italy | 587 adults  Age = 18-52 years  Female = 82.4% Male = 16.4% Non-binary = 1.2% | One item about sharing someone’s sexts without consent from the Sexting Behaviours Quesionnaire^a^. The exact wording of the item is not provided. The item was responded to with a 5-point scale ranging from “never” to “frequently or daily”. | Sending sexts (ns); moral disengagement (+); body esteem (ns) |  |
| Casas et al. (2019) | Longitudinal | Spain | 1,431 secondary school students  Age = 11-18 years  Female = 46.4% Male = 53.6% | One item (“I have forwarded or shared erotic-sexual videos, images or messages of other boys or girls”). Response options were “never”, “rarely”, “occasionally”, “often”, and “frequently”. | Sex/gender (male > female); receiving NCSII (+); previous NCSII perpetration (+); sending sexts (+), receiving sexts (+); prosocial behavior (-) |  |
| Clancy et al. (2019) | Cross-sectional | Australia | 505 adults  Age = 18-55 years  Female = 66.9% Male = 31.5% Unknown = 1.4% | One item (“Have you ever received a sexually explicit image sent via text or mobile app intended for yourself which you subsequently showed/sent to another person?”). Response options were “yes” and “no”. | Sex/gender (ns); age (ns); receiving NCSII (+);NCSII offense supportive cognitions (+); narcissism (+); Machiavellianism (+); psychopathy (+); sexual activity (+); sexual debut age (-) | Receiving NCSII (+); NCSII offense supportive cognitions (mixed); narcissism (ns); Machiavellianism (ns); psychopathy (ns); sexual activity (+) |
| Clancy et al. (2020) | Cross-sectional | USA, UK, Australia, and Canada | 691 adults  Age = 18-35 years  Female = 52.8% Male = 47.2% | One item (“Have you ever received a sexually explicit image sent via text or mobile app intended for yourself which you subsequently showed/sent to another person?”). Response options were “yes” and “no”. | Sex/gender (ns); age (ns); NCSII victimization (+); receiving NCSII (+); sending sexts (+); receiving sexts (+); requesting sexts (+); narcissism (+); Machiavellianism (+); psychopathy (+); sexual activity (+); sexual debut age (-) | Sex/gender (ns); age (ns); NCSII victimization (+); receiving NCSII (+); sendings sexts (ns); receiving sexts (ns); requesting sexts (+); narcissism (+); Machiavellianism (ns); psychopathy (ns); sexual activity (ns) |
| Clancy et al. (2023) | Cross-sectional | Australia, USA, and UK | 2,126 adults  Age = 18-30 years  Female = 55% Male = 45% | One item (“Have you ever received an image-based sext intended for yourself which you subsequently showed/sent to another person?”). Sexts were defined as “sexually explicit images, sent, received, or shared via mobile phone messaging or apps”. Response options were “yes” and “no”. | Sex/gender (ns); NCSII victimization (+); sending sexts (+); receiving sexts (+); receiving unwanted sexts (+); requesting sexts (+); NCSII offense supportive cognitions (mixed); symptoms of anxiety (+); symptoms of depression (ns); psychological distress (+) | Sex/gender (ns); age (ns); NCSII victimization (+); receiving NCSII (+); sending sexts (+); receiving sexts (+); receiving unwanted sexts (+); requesting sexts (ns); NCSII offense supportive cognitions (mixed); symptoms of anxiety (ns); symptoms of depression (ns); psychological distress (ns); sexual activity (ns) |
| Clancy, Klettke, et al. (2021)^c^ | Cross-sectional | USA and Australia | 1,148 adults  Age = 18-29 years  Female = 53% Male = 47% | One item (“Have you ever received an image-based sext intended for yourself which you subsequently showed/sent to another person?”). Sexts were defined as “sexually explicit images, sent, received, or shared via mobile phone messaging or apps”. Response options were “yes” and “no”. | Sex/gender (ns); age (ns); nationality (ns); NCSII victimization (+); receiving NCSII (+); sending sexts (+); receiving sexts (+); receiving unwanted sexts (+); requesting sexts (+); NCSII offense supportive cognitions (+) | Sex/gender (ns); age (ns); nationality (USA > Australia); NCSII victimization (+); receiving NCSII (+); sending sexts (ns); receiving sexts (ns); receiving unwanted sexts (+); requesting sexts (+); NCSII offense supportive cognitions (+) |
| Clancy, Maas, et al. (2021)^c^ | Cross-sectional | USA and Australia | 1,148 adults  Age = 18-29 years  Female = 53% Male = 47% | One item (“Have you ever received an image-based sext intended for yourself which you subsequently showed/sent to another person?”). Sexts were defined as “sexually explicit images, sent, received, or shared via mobile phone messaging or apps”. Response options were “yes” and “no”. | Sex/gender (ns); age (ns); NCSII victimization (+); receiving NCSII (+); sending sexts (+); receiving sexts (+); requesting sexts (+); bullying perpetration (-); bullying victimization (-); symptoms of anxiety (+); symptoms of depression (ns); psychological distress (+); sexual activity (+) |  |
| Gámez‐Guadix et al. (2022) | Longitudinal | Spain | 1,820 adolescents  Age = 12-17 years  Female = 51% Male = 48.2% Non-binary = 0.2% Unknown = 0.5% | Three items (e.g., “You have forwarded a sexual image of someone without their consent”). Response options were “never”, “1 or 2 times”, “3 or 4 times”, and “5 times or more”. | Sex/gender (ns); age (+); NCSII victimization (+); previous NCSII perpetration (+); sending sexts (+); bullying perpetration (+); bullying victimization (+); symptoms of anxiety (+); symptoms of depression (+) |  |
| Gámez‐Guadix et al. (2023) | Cross-sectional | Spain | 1,682 adolescents  Age = 12-18 years  Female = 51.4% Male = 48.5% Unknown = .06% | Three items (e.g., “You have shown someone a sexual image [photos or videos] of another person without their consent”). Limited to experiences in the 12 months prior to the survey. Response options were “never”, “1 or 2 times”, “3 or 4 times”, and “5 times or more”. | Sex/gender (ns); age (+) |  |
| Garcia et al. (2016) | Cross-sectional | USA | 5,805 single adults  Age = 21-75+ years  Female = 48.8% Male = 51.2% | One item (“sent a sexy photo of someone else portraying it as yourself”). Response options were “yes” and “no”. | Sex/gender (male > female); age (ns); sexual orientation (ns) |  |
| Holt et al. (2021) | Cross-sectional | Australia | 1,328 secondary school students (8^th^ graders)^d^  Female = 48.8% Male = 51.2% | One item (“Over the last 12 months, how often have you … shared sexual content (e.g. text, images or videos) of someone else without their consent”). Response options were “never”, “less than weekly”, “about once a week”, “several times a week”, “about once a day”, and “several times a day”. |  | Sex/gender; (ns); self-control (ns); pornography use (+) |
| Hu et al. (2023) | Cross-sectional | Australia, UK, and USA. The nationality of 7.8% of participants is not reported. | 1,688 adults  Age = 18-30 years  Female = 52.7% Male = 47.3% | One item (“Have you ever received an image-based sext intended for yourself which you subsequently distributed to another person [this includes showing or sharing the image]?”). Sexts were defined as “sexually explicit images, sent, received or shared via mobile phone messaging or apps”. Response options were “yes” and “no”. | Sex/gender (ns); age (ns); sending sexts (+); requesting sexts (+); bullying perpetration (+); bullying victimization (ns) |  |
| Karasavva & Forth (2022) | Cross-sectional | Canada | 810 undergraduate students  Age = 16-60 years  Female = 72.7% Male = 27.3% | Eight items about distributing nude or sexual images of someone else, referring to distributing different types of content (e.g., where “they were partially clothed or semi-nude”)^e^. Response options were “yes” and “no”. | NCSII offense supportive cognitions (mixed); narcissism (+); Machiavellianism (ns); psychopathy (+); sadism (+) | Sex/gender (ns); sexual orientation (ns); NCSII victimization (+); NCSII offense supportive cognitions (ns); narcissism (+); Machiavellianism (ns); psychopathy (ns); sadism (+); |
| Klein & Cooper (2019) | Cross-sectional | USA | 812 undergraduate students  Age = 18-22+ years  Female = 63.3% Male = 34.7% | One item about sending or forwarding nude, sexually suggestive, or explicit photos of someone else. The exact wording of the item and response options is not provided. Responses were dichotomized to “never” and “at least once” prior to analysis. |  | Sex/gender (ns); age (ns); sexual orientation (ns); ethnicity/race (mixed); relationship status (ns); level of education (ns); alcohol use (ns); self-esteem (-) |
| Kokkinos et al. (2024) | Cross-sectional | Greece | 2,913 university students  Age = 18-31+ years  Female = 69.6% Male = 30.4% | Eight items (e.g., “How often do you post-sexually or provocative photos or videos where the person pictured is one of the following [a romantic partner, a friend, a stranger], without his or her consent on social media?”). Items were responded to with a 5-point scale ranging from “never” to “frequently or daily”. | Sex/gender (male > female); sexual orientation (ns); sending sexts (+); pressured sexting (+); openness (-); conscientiousness (-); extraversion (ns); agreeableness (-) |  |
| Linares et al. (2021) | Cross-sectional | Spain | 697 undergraduate students  Age = 18-35 years  Female = 78.6% Male = 21.4% | One item (“Sending and/or uploading photos, images and/or videos with intimate or sexual content without permission”). Items were responded to with a 6-point scale ranging from “never” to “always/more than 20 times”. | Sex/gender (ns) |  |
| Longobardi et al. (2024) | Cross-sectional | Italy | 940 young adults  Age = 18-35 years  Female = 79.4% Male = 20.6% | One item (“Have you ever received a sexually explicit image via text message or mobile app intended for yourself which you subsequently distributed to another person (this includes showing or sharing the image)?”). Response options were “yes” and “no”. | Empathy (mixed) |  |
| Lu et al. (2021) | Cross-sectional | USA | 2,199 middle school students  *M*_age_ = 13.53 years  Female = 49.8% Male = 49.6% Unknown = 0.6% | One item (“Have you ever forwarded the nude photo to others without their permission?”). Response options were “yes” and “no”. | Sending sexts (+); pressured sexting (+); receiving unwanted sexts (+); requesting sexts (+); aggressiveness (+); hostility (+); impulsivity (+); symptoms of anxiety (ns); symptoms of depression (+) |  |
| Maes et al. (2023) | Cross-sectional | Belgium | 1,343 secondary school students and adults  Age = 16-30 years  Female = 78.4% Male = 21.6% | Two items (“ever received a sext from a woman/girl and forwarded this sext to others without her permission?” and “ ever received a sext from a man/boy and forwarded this sext to others without his permission?”). Sexts were defined as “sexually explicit pictures or videos”. Response options were “yes” and “no”. | *Female victim* Sex/gender (ns); age (+); sexual orientation (ns); level of education (+); sending sexts (-); empathy (-); narcissism (+); NCSII offense supportive cognitions (+); pornography use (-)  *Male victim* Sex/gender (ns); age (+); sexual orientation (ns); level of education (+); sending sexts (+); empathy (-); narcissism (ns); NCSII offense supportive cognitions (+); pornography use (-) |  |
| Marinho et al. (2023) | Cross-sectional | Portugal | 358 adolescents  Age = 10-20 years  Female = 32% Male = 68% | One item (“Have you ever shared sexually explicit images or videos of other people without their consent?”). Response options were “yes” and “no”. | Emotional victimization (+); sexual victimization (+); physical victimization (+); uncaring (-); callousness (+) |  |
| Martínez-Bacaicoa et al. (2024) | Cross-sectional | Spain | 2,486 adults  Age = 16-79 years  Female = 69% Male = 28.4% Non-binary = 1.9% Unknown = 0.7% | Four items about how many times respondents had perpetrated different types of NCSII (e.g., “You have shown someone sexual content (photos or videos) of another person without their consent”). Limited to experiences in the 12 months prior to the survey. The items were open-ended. | Sex/gender (male > female); age (ns); sexual orientation (non-heterosexual > heterosexual); NCSII victimization (+); sexism (+) |  |
| Morelli et al. (2016) | Cross-sectional | Italy | 715 adolescents and adults  Age = 13-30 years  Female = 71.7% Male = 28.3% | Six items (e.g., “How often have you sent provocative or suggestive pictures or videos of someone you know by SMS/MMS/WhatsApp/ Snapchat without his/her consent?”). Items were responded to with a 5-point scale ranging from “never” to “frequently or daily”. | Sex/gender (male > female); age (-); sexual orientation (ns); sexism (+) |  |
| Morelli et al. (2020) | Cross-sectional | Poland, USA, Italy, Czech Republic, Turkey, Belgium, China, Russia, Ireland, and Uganda | 5,542 adolescents and adults  Age = 13-30 years  Female = 59.8% Male = 40.2% | Eight items (e.g., “How often have you publicly posted sexually suggestive or provocative photos about your partner without his/her consent?”) from the Sexting Behaviors Questionnaire^a^. Limited to experiences in the 12 months prior to the survey. Items were responded to with a 5-point scale ranging from “never” to “always or almost daily”. | Sex/gender (male > female); age (-); sexual orientation (non-heterosexual > heterosexual); sending sexts (+); risky sexting (+); pressured sexting (+); honesty/humility (-); extraversion (ns); agreeableness (ns); conscientiousness (-); openness (-) | Sex/gender (male > female); age (ns); sexual orientation (non-heterosexual > heterosexual); nationality (mixed); honesty/humility (-); extraversion (ns); agreeableness (ns); conscientiousness (-); openness (-) |
| Morelli et al. (2021) | Cross-sectional | Poland, USA, Italy, Czech Republic, Turkey, Belgium, China, Russia, Ireland, and Uganda | 6,093 adolescents and adults  Age = 13-30 years  Female = 60.4% Male = 39.4% Unknown: 0.2% | Eight items from the sexting behaviors questionnaire^a^. No example item is provided. Limited to experiences in the 12 months prior to the survey. Items were responded to with a 5-point scale ranging from “never” to “always or almost daily”. | Sex/gender (male > female); age (-); sending sexts (+); risky sexting (+); pressured sexting (+); narcissism (+); Machiavellianism (+); psychopathy (+) | Sex/gender (male > female); age (-); narcissism (ns); Machiavellianism (+); psychopathy (+) |
| Morelli, Cattelino, et al. (2023) | Cross-sectional | Italy | 760 adolescents  Age = 14-19 years  Female = 52.6% Male = 47.4% | Eight items (e.g., “How often have you privately sent provocative or sexually suggestive photos about your partner without his/her consent?”) from the Sexting Behaviors Questionnaire^a^. Limited to experiences in the 12 months prior to the survey. Items were responded to with a 5-point scale ranging from “never” to “always or almost daily”. | Sex/gender (male > female); age (ns); sending sext (+); risky sexting (+); pressured sexting (+); self-control (ns); empathy (ns); general mental health (ns) | Sex/gender (male > female); age (ns); self-control (ns); empathy (-); general mental health (ns) |
| Morelli, Plata, et al. (2023) | Cross-sectional | Italy and Columbia | 2,931 adults  Age = 18-35 years  Female = 67.6% Male = 32.4% | Eight items (e.g., “How often have you privately sent provocative or sexually suggestive photos about your partner without his/her consent?”) from the Sexting Behaviors Questionnaire^a^. Limited to experiences in the 12 months prior to the survey. Items were responded to with a 5-point scale ranging from “never” to “always or almost daily”. | Sex/gender (male > female); age (ns); sexual orientation (non-heterosexual > heterosexual); nationality (Colombia > Italy) |  |
| Ojeda et al. (2019) | Longitudinal | Spain | 1,736 high school students  Age = 12-16 years  Female = 46% Male = 54% | One item (“I have forwarded or shared erotic-sexual videos, images or messages of other boys or girls”). Response options were “never”, “rarely”, “occasionally”, “often”, and “frequently”. |  | Sex/gender (male > female); age (+); receiving NCSII (+); previous NCSII perpetration (+); sending sexts (ns); receiving sexts (ns); bullying perpetration (mixed) |
| Ojeda et al. (2024) | Cross-sectional | Spain | 1,611 adolescents  Age = 12-15 years  Female = 47.9% Male = 52.1% | Four items (e.g., ‘‘I have forwarded or shared suggestive or sexual text messages featuring girls without their consent”) from the Sexting Behavior and Motives Questionnaire^f^ (note that the scale includes items about images). Limited to experiences in the 12 months prior to the survey. Items were responded to with a 5-point scale ranging from “never” to “daily”. | Sex/gender (male > female); age (ns); moral disengagement (+) |  |
| Patchin & Hinduja (2019) | Cross-sectional | USA | 5,593 middle and high school students  Age = 12-17 years  Female = 49.7% Male = 49.9% Transgender = 0.3% | One item (“(…) ever shared an explicit image that was sent to them with someone else [without the permission of the sender]). Response options were “yes” and “no”. | Sex/gender (male > female); age (curvilinear); sexual orientation (non-heterosexual > heterosexual); ethnicity/race (ns) |  |
| Peskin et al. (2013) | Cross-sectional | USA | 1,034 high school students  Age = 14-19 years  Female = 62.6% Male = 37.4% | One item (“[…)]ever shared a nude or semi-nude picture/video with someone other than the one(s) it was originally meant for”). Response options are not provided. Analyzed as a dichotomous variable. | Sex/gender (male > female); ethnicity/race (ns) |  |
| Powell et al. (2019) | Cross-sectional | Australia | 4,053 adolescents and adults  Age = 16-49 years  Female = 56.7% Male = 43.3% | Eight items referring to distributing different types of content (e.g., “Where they were partially clothed or semi-nude”) since the age of 16. Response options were “yes” and “no”. | Sex/gender (male > female); sexual orientation (non-heterosexual > heterosexual) |  |
| Powell et al. (2022) | Cross-sectional | UK, Australia, and New Zealand | 6,109 adolescents and adults  Age = 16-49 years  Female = 52.1% Male = 47.9% | Eight items referring to distributing different types of content (e.g., “Where they were partially clothed or semi-nude”) since the age of 16. Response options were “yes” and “no”. | Sex/gender (male > female); nationality (New Zealand > UK and Australia) |  |
| Ruvalcaba & Eaton (2020) | Cross-sectional | USA | 3,044 adults  Age = 18-97 years  Female = 53.8% Male = 46.2% | One item (“Have you ever knowingly shared a sexually explicit image or video of someone else without his/her consent?”). Response options were “no, never”, “yes, once”, and “yes, more than once”. | Sex/gender (male > female); age (-) |  |
| Said & McNealy (2023) | Cross-sectional | USA | 713 college students^d^  Female = 66% Male = 32% Non-binary < 1.5% | Three items about ever showing, sending, and posting explicit photos or videos of others without their permission. Exact wording is not provided. Response options were “yes” and “no”. |  | Sex/gender (ns); age (ns); ethnicity/race (ns); relationship status (ns); NCSII victimization (+); sending sexts (ns); receiving unwanted sexts (+); risky sexting (ns) |
| Schokkenbroek et al. (2023) | Cross-sectional | Belgium | 169 adults  Age = 18-76 years  Female = 73.4% Male = 26.6% | One item (“we asked respondents how often they had ever seen or received sexual pictures or messages from someone else without the original sender’s knowledge”). The item was responded to with a 4-point scale ranging from “never” to “often”. | Sex/gender (ns); age (ns); receiving NCSII (+); NCSII offense supportive cognitions (+) | Sex/gender (ns); age (ns); receiving NCSII (+); NCSII offense supportive cognitions (ns) |
| Sesar et al. (2021) | Cross-sectional | Croatia and Bosnia-Herzegovina | 707 adults  Age = 18-39 years  Female = 80% Male = 20% | Two items regarding forwarding “semi-nude or nude” (1) photos and (2) videos. The exact wording of the items not provided. Items were responded to with a 5-point Likert scale ranging from “never” to “regularly or every day”. | *Forwarding photographs* Sex/gender (male > female); self-esteem (-); body esteem (mixed)  *Forwarding photographs* Sex/gender (male > female); self-esteem (ns); body esteem (ns) | Sex/gender (male > female); relationship status (ns); self-esteem (ns); body esteem (mixed) |
| Seto et al. (2023) | Cross-sectional | USA | 996 adolescents  Age = 9-17 years  Female = 60.3% Male = 35.3% Unknown = 4.4% | One item (“have you ever shared a nude or nearly nude photo or video of someone else without that person's knowledge online?”). Information about response options is not provided. | Sex/gender (ns); age (ns); sexual orientation (ns); ethnicity/race (ns); area of residence (urban/suburban > rural); NCSII victimization (+); sending sexts (+); receiving sexts (+); NCSII offense supportive cognitions (+); dating app use (+) | Sex/gender (ns); age (ns); sexual orientation (ns); race/ ethnicity (ns); area of residence (urban/suburban > rural); NCSII offense supportive cognitions (+); dating app use (ns) |
| Silva et al. (2020) | Cross-sectional | Brazil | 202 university students (18 years and older)^d^  Female = 63.9% Male = 36.1%) | One item about sending partner’s intimate images. The exact wording of the item and response options is not provided. | Sex/gender (male > female); age (ns); sexual orientation (ns); ethnicity/race (ns); relationship status (ns); alcohol use (ns); drug use (+); empathy (mixed); pornography use (ns) |  |
| Smith et al. (2018) | Cross-sectional | Canada | 398 high school students  Age = 14-18 years  Female = 56.4% Male = 43.6% | One item about publishing a “photo of sexual nature” of their partner online. The exact phrasing of the item and response options is not provided. | Psychological distress (ns); self-esteem (ns) |  |
| Sparks et al. (2023) | Cross-sectional | Canada | 541 adults  Age = 18-25 years  Female = 68.6% Male = 31% Unknown = 0.4% | Six items (e.g., “how often have you uploaded someone’s sexually explicit nude—either partial or full—images or videos online or to social media without consent?”). Information about response options is not provided. Responses were dichotomized prior to analysis. | NCSII victimization (+)  *Victim-perpetrators vs. no NCSII experience* impulsivity (+); alcohol use (ns); drug use (+); NCSII offense supportive cognitions (+); Machiavellianism (+); narcissism (+); psychopathy (+); sadism (ns); |  |
| Strassberg et al. (2013) | Cross-sectional | USA | 606 high school students^d^  Female = 46% Male = 54% | Information about the number items, exact phrasing of the item(s), and response option(s) is not provided. | *Forwarding received sexually explicit image* Sex/gender (ns)  *Forwarding sexually explicit image taken of someone else* Sex/gender (male > female) |  |
| Strassberg et al. (2014) | Cross-secto | USA | 1,130 college students^d^  Female = 59.2% Male = 40.8% | Information about the number items, exact phrasing of the item(s), and response options is not provided. | Sex/gender (male > female) |  |
| Strassberg et al. (2017) | Cross-sectional | USA | 622 high school students^d^  Female = 46% Male = 54% | Information about the number items, exact phrasing of the item(s), and response options is not provided. | Sex/gender (ns) |  |
| Van den Eynde et al. (2023) | Cross-sectional | Belgium | 8,439 secondary school students  *1^st^ year students* *M*_age_ = 13.2 years  Female = 50.7% Male = 49.3%  *2^nd-^3^rd^ year students* *M*_age_ = 16.3 years Female = 53.9% Male = 46.1% | One item (“Over the past 12 months, have you ever disseminated sexually explicit photos or videos of someone else via the Internet or your phone (without that person’s consent)?“). Response options were “no/never”, “1 time”, “2 times”, “3 times”, and “more than 3 times”. Responses were dichotomized prior to analysis. | NCSII victimization (+) | *1^st^ year students* Sex/gender (ns); age (+); NCSII victimization (+)  *2^nd^ and 3^rd^ year students* Sex/gender (ns); age (-); NCSII victimization (+) |
| Van Ouytsel et al. (2019) | Cross-sectional | Belgium | 3,109 adolescents  Age = 12-15 years  Female = 53.5% Male = 46.5% | One item (“Ever forwarded a sext themselves without knowledge of the person who made the image”). Responded to with a 5-point scale ranging from “never” to “weekly”. Responses were dichotomized prior to analysis. |  | Sex/gender (ns); age (+); sexual orientation (ns) |
| Van Ouytsel et al. (2021) | Cross-sectional | Belgium | 1,306 middle and high school students  *M*_age_ = 15 years  Female = 50.5% Male = 49.5 | One item (“In addition to the receiving of non-consensual sexting images, respondents were also asked if they had ever forwarded an image to others without this person knowing about it”). Information about response options is not provided. | Sex/gender (ns); age (+); sexual orientation (ns) |  |
| Wachs et al. (2021) | Cross-sectional | USA | 2,506 high school students  Age = 13-16 years  Female = 50% Male = 50% | Six items (e.g., “How often have you sent sexually suggestive or provocative photos/videos about your partner by SMS/MMS/ WhatsApp/Snapchat without his/her consent?”) from the Sexting Questionnaire^a^.Items were responded to with a 5-point scale ranging from “never” to “always or almost daily”. | Sending sexts (+); pressured sexting (+); symptoms of depression (+) | Sex/gender (male > female); sexual orientation (ns); ethnicity/race (ns) |
| Walker et al. (2021) | Cross-sectional | England | 391 adults  Age = 18-25 years  Female = 82.1% Male = 17.9% | Information about the number of items (e.g., “Have you ever shared sexy messages with other individual(s) without the consent of the sender?”) is not provided. Response options were “yes” and “no”. | Sex/gender (ns); NCSII victimization (+) |  |

*Note.* NCSII = non-consensual sharing of intimate images. Statistically non-significant findings are reported as “ns”. Inconsistent results for two or more dimensions of to the same construct (e.g., NCSII offense-supportive cognitions) are labelled “mixed”.

^a^ Developed by Morelli et al. (2016)

^b^ Findings reported by Clancy, Klettke et al. (2021) and Clancy, Maas et al. (2021) are based on the same sample

^c^ Developed by Clancy et al. (2020)

^d^ Neither age range nor mean was reported.

^e^ Developed by Powell et al. (2019)

^f^ Developed by del Rey et al. (2021)

## Table C2 *Summary of quality assessment of quantitative studies (*n *= 55)*

|  | Assessment | | | |
| --- | --- | --- | --- | --- |
| Methodological criteria | Yes | No | Can’t tell |  |
| Is the sampling strategy relevant to address the research question? | 44 | 1 | 10 |  |
| Is the sample representative of the target population? | 3 | 32 | 20 |  |
| Are the measurements appropriate? | 52 | 1 | 2 |  |
| Is the risk of nonresponse bias low? | 2 | 7 | 46 |  |
| Is the statistical analysis appropriate to answer the research question? | 54 |  | 1 |  |

*Note.* Quality criteria for quantitative descriptive studies, as outlined by the Mixed Methods Appraisal Tool (Hong et al., 2018).

**References (not included in article)**

Morelli, M., Bianchi, D., Baiocco, R., Pezzuti, L., & Chirumbolo, A. (2016). Sexting, psychological distress and dating violence among adolescents and young adults. *Psicothema*, *28*, 137–142. <https://doi.org/10.7334/psicothema2015.193>

del Rey, Rr, Ojeda, M., Casas, J. A. (2021). Validation of the Sexting Behavior and Motives Questionnaire (SBM-Q). *Psicothema*, *33*(2), 287-295. <https://doi.org/10.7334/psicothema2020.207>

# Appendix D Characteristics and Quality Assessment of Quantitative Studies

## Table D1 *Key characteristics and findings of qualitative studies (*n *= 13)*

| Author(s) | Design | Setting(s) | Sample(s) | Analytical approach | Key findings |
| --- | --- | --- | --- | --- | --- |
| Hall & Hearn (2019) | Digital ethnography | MyEx.com | 4418 posts by perpetrators explicitly seeking revenge | Discourse analysis, drawing on theories of gender-based violence, affordances, masculinities, and manhood acts | This study shows how ‘manhood acts’ are invoked by posters on revenge pornography websites to account for their decision to share private intimate images of others without their consent. Victim blaming was present in all posts, with underlying themes such as power, control, and heterosexuality. |
| Hall et al. (2022) | Digital ethnography | thecandidzone.com | One post, incl. comments from forum users | Thematic analysis, drawing on theories of masculinities, homosociality, gender-based violence, craftsmanship, and symbolic creativity | This study reveals that both posters and respondents on upskirting websites perceive this activity as an artistic and technical practice, often providing one another with advice and guidance on achieving the desired shots (craftsmanship). Homosociality and craftmanship were the two key themes identified. |
| Harder (2021) | Semi-structured interviews | Denmark | 25 young adults (ages 18-25 years), of which 13 were men, 11 were women, and 1 was non-binary | Thematic analysis, drawing on theories of emotion work and bystander responses | This study indicates that bystanders perceive NCSII situations as awkward, uncomfortable, and ethically troubling. Instead of directly intervening, they cope with their conflicting emotions using different strategies such as ‘surface acting’, ‘deep acting’ and the use of cropping technologies. |
| Hearn & Hall (2019) | Digital ethnography | MyEx.com | Posts by perpetrators^a^ | Discourse analysis, drawing on theories of gender and power | This study highlights how gender and sexuality are invoked in the motivations and justifications for revenge pornography. The authors state that there are clear differences between discursively constructed gender/sexual orientations and genres of the posts and posters. |
| Hunehäll Berndtsson (2022) | Focus groups and semi-structured interviews | Sweden | 18 secondary school students (ages 15-16 years), of which 14 were boys and 4 were girls | Thematic analysis, drawing on theories of heteronormativity and (male) vulnerability | This study highlights that Swedish teenage boys are frequently exposed to various nonconsensual intimate image sharing practices. However, heteronormative norms and traditional notions of heterosexual masculinity prevent them from openly discussing, understanding, or addressing instances of digital sexual harassment, resulting in a sense of isolation. |
| Johnsonbaugh (2021) | Semi-structured interviews | USA | 55 college students (ages 18-25 years), of which 30 were women and 25 were men | Thematic and typology analysis, drawing on interaction ritual theory | This study reveals that men often share images of women with their peers to showcase sexual prowess and gain social validation, while women share images of men with their peers as a way to cope with unwelcome sexual advances and seek support. |
| Naezer & van Oosterhout (2021) | Semi-structured interviews | Netherlands | 21 young perpetrators, victims, and bystanders of nonconsensual image sharing (ages 15-21 years), of which 13 were girls and 8 were boys | Thematic analysis, drawing on theories of social norms, gender dynamics, and sexual taboos | This study demonstrates that non-consensual image sharing is deeply connected to social norms regarding gender and sexuality. The research identifies multiple motives for non-consensual image sharing, including revenge, tension release, reinforcing friendships, discussing sexuality, regulating others' sexual behavior, and gaining popularity. |
| O’Rourke & Haslop (2024) | Focus groups and semi-structured interviews | United Kingdom | 40 young men (ages 18-25 years) | Thematic analysis, drawing on theories of masculinity and homosociality | This study elucidates how digital communication technologies are used by young men in the UK to facilitate and mediate hetero-masculine homosociality. Key themes were “camaraderie, solidarity, and emotional intimacy”, “hetero-masculinity, misogyny and gendered hierarchies”, and “changing attitudes to misogynistic forms of homosociality”. |
| Otteren & Gynnhild (2021) | Digital ethnography | A Norwegian branch of an unnamed global online community | Posts connected to 20 different topics on the discussion board^a^ | Classic grounded theory | This study develops the theory of remote female fixation. The authors argue that that the main concern of the forum users is to manage their distant fixation on female nudity, so they engage in collective sharing activities online. |
| Rasmussen & Søndergaard (2021) | Digital ethnography, fieldwork, and interviews | Denmark  Various social media (e.g., Instagram, Reddit, Discord), and other unnamed online communities | 21 vocational school students^b^, of which 18 were boys and 3 were girls  20 pages of observation notes and 300 screenshots of user interactions, norms and contexts, and digital architectures (etc.) | Discourse analysis, drawing on theories of gender norms and dynamics, masculinity, and boundary work | This study highlights how men use sexualized female bodies to negotiate boundaries of masculinity, gendered positioning, and intimacy. Their actions focus on performing masculinity and maintaining gender inequality. |
| Ringrose et al. (2013) | Focus groups, semi-structured interviews, and digital ethnography | United Kingdom | 35 secondary school students (ages 12-15 years)^c^ | Thematic analysis^d^, drawing on feminist theories of gender, sexualization, objectification, and moral visual representation | This study elucidates how young people’s discussions of intimate image sharing practices reinforce gendered sexual morals and blurs distinctions between pleasurable and coercive interactions. |
| Semenzin & Bainotti (2020) | Digital ethnography | Telegram | User posts and interactions in 50 channels and groups^a^ | Ethnographic content analysis drawing on theories of hegemonic masculinity and homosociality | This study suggests that Telegram's affordances, such as anonymity, weak regulation, and the possibility of creating large male communities, play a significant role in facilitating and normalizing the non-consensual dissemination of intimate images. The platform's features consist as “gendered affordances” that orient male participants’ harassment behaviors and contributes to the reinforce hegemonic masculinity. |
| Storrod (2024) | Focus groups and digital ethnography | United Kingdom  Various social media (e.g., Youtube, Snapchat, X) | 240 young people (ages 12-15 years), of which 125 were girls and 115 were boys  50 social media profiles belonging to 46 young men linked to gangs | Classic grounded theory | This study elucidates experiences of and perspectives on technology-facilitated sexual violence among young people in and around gangs in London. Findings emphasize that gender plays a crucial role in gang dynamics, and thereby influences experiences of (e.g.) sexting, getting exposed, and being sexed-in. |

^a^Number of posts were not reported

^b^Age range was not reported

^c^Gender distribution was not reported

^d^Ringrose et al. (2013) do not state a particular analysis method, but the results are structured around themes and the approach was therefore labelled as thematic analysis

## Table D2 *Summary of quality assessment of qualitative studies (*n *= 13)*

|  | **Assessment** | | |
| --- | --- | --- | --- |
| **Methodological criteria** | **Yes** | **No** | **Can’t tell** |
| Is the qualitative approach appropriate to answer the research question? | 11 |  | 2 |
| Are the qualitative data collection methods adequate to address the research question? | 11 |  | 2 |
| Are the findings adequately derived from the data? | 7 |  | 6 |
| Is the interpretation of results sufficiently substantiated by data? | 11 | 1 | 1 |
| Is there coherence between qualitative data sources, collection, analysis and interpretation? | 8 | 4 | 1 |

*Note.* Quality criteria for qualitative studies, as outlined by the Mixed Methods Appraisal Tool (Hong et al., 2018).
